# New Zn3Mg-xY Alloys: Characteristics, Microstructural Evolution and Corrosion Behavior

**DOI:** 10.3390/ma14102505

**Published:** 2021-05-12

**Authors:** Catalin Panaghie, Ramona Cimpoeșu, Bogdan Istrate, Nicanor Cimpoeșu, Mihai-Adrian Bernevig, Georgeta Zegan, Ana-Maria Roman, Romeu Chelariu, Alina Sodor

**Affiliations:** 1 Faculty of Materials Science and Engineering, “Gh. Asachi” Technical University from Iasi, 700050 Iasi, Romania; catalin.panaghie@student.tuiasi.ro (C.P.); nicanor.cimpoesu@tuiasi.ro (N.C.); mihaibernevig@gmail.com (M.-A.B.); ana-maria.roman@academic.tuiasi.ro (A.-M.R.); chelariu@tuiasi.ro (R.C.); 2 Faculty of Mechanical Engineering, “Gh. Asachi” Technical University from Iasi, 700050 Iasi, Romania; bogdan_istrate1@yahoo.com; 3 Faculty of Dental Medicine, “Grigore T. Popa” University of Medicine and Pharmacy, 700115 Iasi, Romania; alinasodor@yahoo.com

**Keywords:** biodegradable alloy, ZnMgY, corrosion, immersion test, 10xDPBS, SEM, XRD, EDS

## Abstract

Zinc biodegradable alloys attracted an increased interest in the last few years in the medical field among Mg and Fe-based materials. Knowing that the Mg element has a strengthening influence on Zn alloys, we analyze the effect of the third element, namely, Y with expected results in mechanical properties improvement. Ternary ZnMgY samples were obtained through induction melting in Argon atmosphere from high purity (Zn, Mg, and Y) materials and MgY (70/30 wt%) master alloys with different percentages of Y and keeping the same percentage of Mg (3 wt%). The corrosion resistance and microhardness of ZnMgY alloys were compared with those of pure Zn and ZnMg binary alloy. Materials were characterized using scanning electron microscopy (SEM), energy dispersive spectroscopy (EDS), X-ray diffraction (XRD), linear and cyclic potentiometry, and immersion tests. All samples present generalized corrosion after immersion and electro-corrosion experiments in Dulbecco solution. The experimental results show an increase in microhardness and indentation Young Modulus following the addition of Y. The formation of YZn12 intermetallic phase elements with a more noble potential than pure Zinc is established. A correlation is obtained between the appearance of new Y phases and aggressive galvanic corrosion.

## 1. Introduction

Certain metals have begun to attract great interest over the years and started to be used in various medical applications due to their good mechanical properties, formability, and wear resistance in time [1,2,3,4,5]. These metals can be characterized by three words: ‘biodegradable’, ‘bioabsorbable’, and ‘bioresorbable’. Materials such as magnesium (Mg), iron (Fe), and zinc (Zn) were studied and accepted as good implant material [6,7,8]. From these three materials, zinc (Zn) is the last studied [2] and is considered promising for more reasons. Researchers established that some issues related to Mg and Fe can be solved using Zn [9,10].

Zinc is an ideal candidate for its good degradation rate and acceptable biocompatibility [11]. Moreover, zinc is the second most abundant trace element in the human body and influences important metabolic processes, and it regulates the cell cycle [12]. Zinc plays an important role in the prevention of heart disease, for example, maintaining the integrity of endothelial cells [13], simulating the proliferation of endothelial cells through increasing the levels of endogenous basic fibroblast growth factor [14], and can prevent further damage caused by ischemia and infarction [15]. Pure zinc stents have been tested in vivo on rabbits and the results have been promising in terms of biodegradability, with no major signs of inflammation or thrombosis formation.

However, the mechanical properties of zinc are below the standards required for metals used in medical applications such as vascular stents. To improve the mechanical properties of pure zinc, it is necessary to add alloying elements with properties that should compensate for the disadvantages of zinc. Alloying elements such as Ca, Mg, Mn, Ge, and Cu is standard practice, and they have been used and studied for developing biodegradable Zn-based alloys with mechanical properties significantly improved [16,17,18,19,20].

The Zn-Mg biodegradable alloy system was studied by many researchers. Mg has 0.1 wt% solubility in Zn or less at 364 °C. As cast Zn-Mg alloys contain Zn primary dendrites and lamellar eutectic mixture of Zn and Mg2Zn11 confirmed by phase diagram and metallographic observations [21]. By adding the Y element in different mass percentages of the Zn-Mg system, the first days of immersion improvement of corrosion resistance and mechanical properties are considered.

In this article, preliminary results obtained on a new alloy (ZnMgY) were analyzed to appreciate the corrosion resistance and hardness variation compared with pure Zn and Zn3Mg materials. Zn alloys are, nowadays, appreciated as a promising material for medical biodegradable applications since their corrosion rate is between magnesium (too big corrosion rate-degradation) and iron (small corrosion rate-degradation), besides other benefic biological reactions of Zn [22,23,24,25,26].

## 2. Materials and Methods

### 2.1. Materials

The samples were obtained using high purity Zn and Mg and master alloy MgY (70/30 wt%) acquisitioned from Hunan China Co., Hunan, China [27], maintain for 10 min at T = 450 °C in a classical induction furnace with Ar atmosphere (~0.75 atm), Inductro, Bucharest, Romania. The experimental set consists of five samples, respectively, pure Zn, ZnMg, and three samples based on ZnMgxY with x = 0.4, 0.5, and 0.6. Experimental ingots (100 g) were obtained from the next material quantities: for alloy Zn3Mg0.4Y we use 96.6g pure Zn, 1.35 g MgY, and 2.05 pure Mg; for Zn3Mg0.5Y we use 96.5 g pure Zn, 1.68 g MgY, and 1.825 pure Mg; for Zn3Mg0.6Y we use 96.0 g pure Zn, 2.6 g MgY, and 1.6 pure Mg. Zinc loss by volatilization was achieved by keeping a reduced temperature of overheating of the metal bath and dilution of the alloying elements. The samples were five times re-melted to obtain proper chemical and structural homogeneity and to reduce the voids and micro-cracks from the melting process. To establish the re-melting effect, we perform surface state analyzes (nondestructive test: NDT) using fluorescent penetrant liquids.

All samples were subjected to nondestructive testing. The samples were cleaned in an ultrasound machine Geti (Tipa, Sadovacity, Czech Republic) before penetrant testing. Fluorescent penetrant testing provides a means of detecting surface-opening discontinuities such as porosity, cracks, or inclusions. The method used is hydrophilic post-emulsification. In this scope, we used an ultra-high sensitivity level 4 penetrant solution and a hydrophilic emulsifier in 5% concentration. A dry developer was used for a better contrast that amplifies the location of pores. Steps used for each stage were as follows: penetrant dwells time 20 min, emulsifier time 2 min, and developer time 10 min. The parts were inspected under UV light with an intensity of 3800 µW/cm^2^ measured at 38 cm distance from the lamp’s bulb.

### 2.2. Corrosion Behavior Analysis

The corrosion behavior was evaluated through immersion and electro-corrosion tests. In all chemical experiments, we use a Dulbecco’s Phosphate Buffered Saline solution (SAFC Bioscience LTD, Hampshire, UK.) (without calcium or magnesium, code: 17-515Q): 10xDPBS (chemical composition: KCl:0.2; KH_2_PO_4_: 0.2; NaCl: 8.0 and NaHPO_4_(anhydrous): 1.15).

Biodegradable materials can be used in melted, heat-treated or plastic deformed state, based on the application requirements; in this article, we analyze the melted state behavior of the samples. Immersion experiments (24, 48, and 72 h) were realized in a thermal-controlled equipment at 37 °C for different periods with regulation of the pH value at 24 h. The mass variation was determined by a Partner analytical balance, Partner Co., Bucharest, Romania, after the immersion tests and after cleaning in an ultrasound bath with an alcohol –based solution.

For electro-corrosion analyze a VoltaLab-21 potentiostat (Radiometer, Copenhagen, Denmark) was used to investigate the linear and cyclic potentiometry in 10xDPBS electrolyte solution. Tests were made using a three-electrode cell. A calomel-saturated electrode was used as a reference electrode and as an auxiliary electrode a platinum one. For the working electrode, all samples were inserted in Teflon support to expose a 0.80 cm^2^ area to the electrolyte. The electrolyte was continuously mixed with a magnetic stirrer to eliminate the bubbles due to H_2_ elimination even that in the case of Zn-alloys the release of H_2_ is much lower than Mg-alloys. To obtain the corrosion potential (Ecorr) and corrosion current (Jcorr) characteristics for each sample, we plot the results as current density (mA/cm^2^) function of potential (V). The corrosion current density, Jcorr, was determined using two equations by extrapolation of the Tafel lines: a complete polarization curve consisting of a cathodic part and an anodic part. The cathodic portion of the polarization curve contains information concerning the kinetics of the reduction reactions occurring for a particular system. The corrosion potential is deduced from the intersection of the anodic and cathodic Tafel slopes. The tests were made at room temperature (RT) (23 °C), and the potential records registered (linear plots were registered at a scan rate of 1 mV/s and the cyclic plots at a scanning rate of 10 mV/s). A scan rate of 1mV/s and a potential scan of approximately ±350 mV about Ecorr is generally required to made reasonably accurate extrapolation. The tests were repeated five times to achieve proper repeatability of the results.

Surface and microstructure were investigated using scanning electron microscopy, VegaTescan LMH II SEM (VegaTescan, Brno—Kohoutovice, Czech Republic), Secondary Electrons (SE) detector (VegaTescan, Brno—Kohoutovice, Czech Republic), electron gun supply: 30 kV, high pressure, 15.5 mm distance between the electron gun and the sample. Chemical composition insights were taken with an EDS detector, Bruker X-Flash, Mannheim, Germany using automatic and element list mode, in Point analysis a surface of 0.05 µm^2^ is investigated. XRD experiments were realized on Expert PRO MPD equipment, Panalytical (XRD, Panalytical, Almelo, The Netherlands model, with copper—X-ray tube (Kα-1.54°). In processing the obtained data, diffractogram patterns were analyzed using the Highscore Plus software (2.2, Panalytical, Almelo, The Netherlands). Samples with area of 50 mm^2^ were scanned in transmission mode as polished discs under the following parameters: 2 theta: 10°–1100°, step size: 0.13°, time/step: 51 s, and a scan speed of 0.065651°/s.

### 2.3. Microhardness Investigation

Microhardness experiments were realized using laboratory equipment CETR UMT-2 Tribometer (Universal Micro-Materials Tester), from the Tribology laboratory of the Mechanical Engineering Faculty from Iasi, Romania. The microindentation evaluation. For tests, we prepared rectangular samples with plan parallel faces with (L:w:t) 10 cm × 2 cm × 1 cm dimensions. The indentations (neatness of the results was supported by five determinations for each sample at 0.25 mm distance one of the others) were made with Rockwell diamond tip (120° opening angle). Dimensions of Rockwell’s tip indenter are a radius of 200 ± 5 μm; angle 120° ± 0.30° and a standard deviation from the median line of ±2 μm. Using this equipment, we managed to evaluate and compare the values of indentation Young modulus (GPa), hardness (Gpa), and contact stiffness (N/µm) based on the determination of the maximum load, (N), maximum displacement, (µm), contact depth, (µm) and contact area, (µm^2^). The method is based on a gradual increase in the indentation load from 0 to 10 N and release to the starting value. The electro-electronic part based on a capacitive sensor along with the load sensor permits the realization of a typical indentation diagram (load-depth). UTM Viewer program (CETR, Campbell, CA, USA) translates data files produced with the UTM testing software (2.16) (CETR, Campbell, CA, USA) into a graphical display for analysis [28].

## 3. Results

Preliminary results from the obtaining and characterization of a new alloy (ZnMgY) with possible medical applications are presented.

### 3.1. Preliminary Results of the Samples

**Figure 1:**
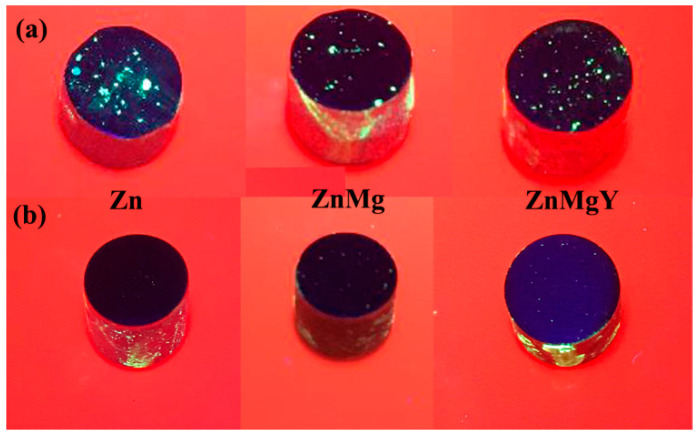
NDT analysis of melted Zn, ZnMg, and ZnMgY alloy (**a**) first melt sand (**b**) after five re-melts.

Experimental materials were NDT tested to establish de effects of the melting operation. The sample surface presents important differences before and after five times re-melting of the alloys (Figure 1), fact that strongly recommends this operation if the classical melting process is used to obtain Zn-alloy. In Figure 1a, the penetrant liquids reveal some surface defects like pores, micro-cracks, or inclusions that will influence alloy properties like corrosion resistance. After a re-melting operation, we obtain a better quality of material surface with less and smaller defects, as can be observed in Figure 1b.

**Figure 2:**
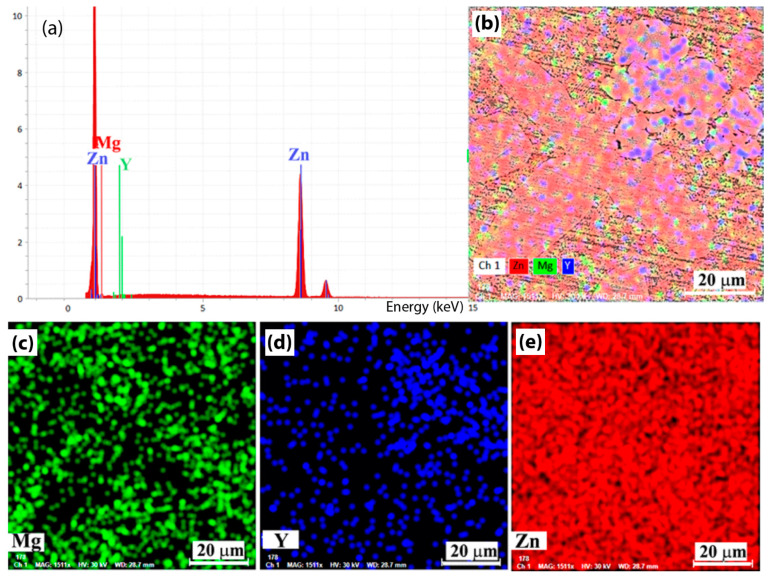
Chemical analysis of experimental alloy (**a**) energy spectrum, (**b**) Zn,MgY elements distribution, (**c**) Mg, (**d**) Y, and (**e**) Zn distributions.

In this article, we will focus on analysis of the ZnMgY alloy, a less researched system than binary ZnMg material in comparison with pure Zn and Zn3Mg, also cast samples. Figure 2 shows the qualitative results of chemical analysis of ZnMgY alloy through the energies of dispersive characteristic X-ray of the new material with the identification of main components: Zn, Mg and Y. A simple distribution of the elements (Figure 2b–e) shows that different chemical composition compounds are present in the alloy. Beside a compound with more Y, located on the right corner of the investigated area, the elements appear in a homogeneous spread.

From elemental distributions (Figure 2c–e), we observe that Y is spread on the entire area, with different intensities, and magnesium participates at different phases with areas with a low or at all contributions.

Analyzing binary diagrams, Mg–Zn, Mg–Y and Y–Zn published in ASM Handbook, vol. 3, Alloys and Phase Diagrams [29], we identified the possibility of formation of different binary compounds such as Mg_2_Zn_11_ (for 93.7 wt% Zn), MgZn_2_ (for 84–84,6 wt%), Mg_2_Zn_3_(for 80.1 wt% Zn), MgZn (for 74 wt% Zn) or Mg_7_Zn_3_(for 53.6 wt% Zn). No compounds Mg–Y with more than 30 wt% Y were identified on our analyses. From Y–Zn binary diagram, the formation of the following compounds was identified: α and β YZn_2_ (for 59.6 wt% Zn), YZn_3_ (for 69 wt% Zn), Y_3_Zn_11_(for 73 wt% Zn), Y_13_Zn_58_ (for 76,7 wt% Zn), YZn_5_ (for 76.6 wt% Zn), Y_2_Zn_17_ (for 86.2 wt% Zn), and YZn_12_ (for 89.8 wt% Zn).

Using a ternary diagram of Mg–Zn–Y predicted from the energy’s formation [30], eleven stable compounds were determined in this alloy: Mg_21_Zn_25_, MgZn_2_, Mg_2_Zn_11_, Mg_24_Y_5_, Mg_2_Y, MgY, Zn_12_Y, Zn17Y2, Zn3Y, ZnY, and MgZnY. Part of these compounds, based on thermodynamic conditions and chemical composition, formed in alloy and were identified by XRD analysis.

**Figure 3:**
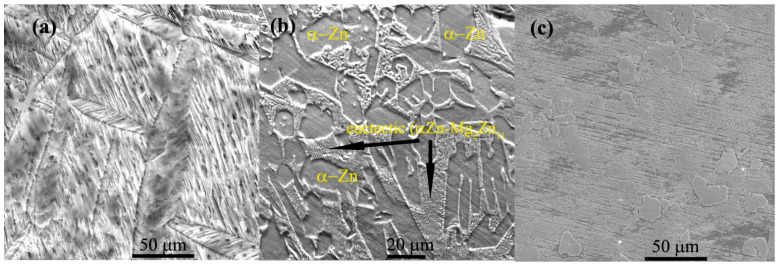
Structural aspects of the samples before corrosion tests (**a**) Zn, (**b**) ZnMg, and (**c**) ZnMgY.

**Figure 4:**
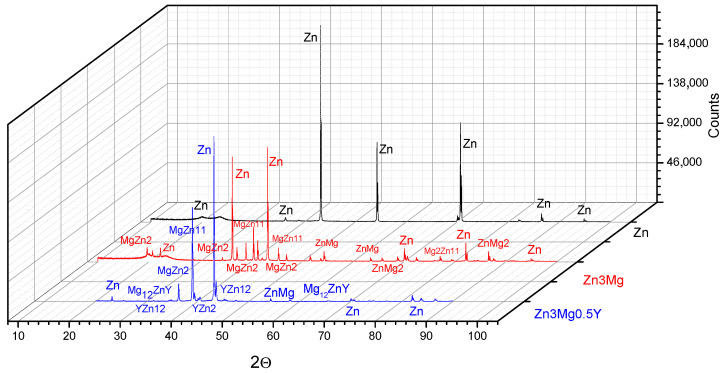
XRD results of Zn, ZnMg, and ZnMgY materials.

Structurally, five areas were identified as having different shapes denoted x1–x5, the figure from Table 1 and Figure 3c, and their chemical composition was determined and presented in Table 1. The alloy was made of mainly pure Zn and master alloy MgY. For x1 point (a spot with 90 nm diameter), a Zn–Y compound is obtained (Table 1), which was identified also with XRD equipment (Figure 4) that has an 11.5 atomic report between Zn and Y that corresponds to YZn12 phase (chemical formula: Zn24.00Y2.00). Here, the affinity of Y from MgY master alloy was stronger for Zn and the formation of YZn12 compound. Point x2 is situated on a differently morphological compound, a complex one ZnMgY. The third area (point 3) represents the matrix, a solid solution of Zn with more Mg and Y dissolved in. The fourth area analyzed, x4, presents a reduced percentage of Mg and a near proposed percentage of Y in composition with zinc in rest. The last compound, point x5, is a ZnMg without Y element. No MgY compounds (introduced in the metallic bath as master-alloy) were observed on the structure after melting and pouring operations.

After remelting and pouring of the materials in a metallic casting form, experimental ingots were mechanically prepared by eliminating the edges and cutting cylindrical samples, mechanical grinding, and chemical etching with the microstructures presented in Figure 3. In all corrosion resistance tests performed in this article, we used ground and polished samples without chemical etch having a structural state near the ones presented in Figure 3. Chemical composition was determined for each ingot through five determinations and the values presented in Table 2 with standard deviations for each element and EDS detector error.

The samples: Zn_3_Mg_0.4_Y, Zn_3_Mg_0.5_Y and Zn_3_Mg_0.6_Y were investigated and compared with pure Zn and Zn_3_Mg. In Figure 3, structural aspects of the samples present different orientations of the grains in the case of pure Zn and a dendritic structure of ZnMg alloy. Microstructural investigations of ZnMg alloy in cast state present α-Zn grains (Figure 3b) and a eutectic (consisting of α–Zn and intermetallic Mg_2_Zn_11_ phase) located along the grain boundaries [31]. All samples present good structural homogeneity, no high quantities of inclusions on the grain boundaries. Figure 3c presents a good distribution of the ZnY and ZnMg compounds in ZnMgY alloy.

Using a ternary diagram of Mg–Zn–Y predicted from the energy’s formation (ternary), eleven stable compounds were determined in this alloy: Mg_21_Zn_25_, MgZn_2_, Mg_2_Zn_11_, Mg_24_Y_5_, Mg_2_Y, MgY, Zn_12_Y, Zn_17_Y_2_, Zn_3_Y, ZnY, and MgZnY. Part of these compounds, based on thermodynamic conditions and chemical composition, formed in alloy and identified by XRD analysis. Besides the usual compounds determined for ZnMg alloy and main peaks of pure Zn, we determined the presence of the YZn_12_ (Figure 4) compound confirming the identification made through EDS detector on Table 1, point x1. For this compound, we identify few 2θ peaks as 40.509 (Int:100, h k l:3 2 1) and 45.497 (Int: 80, h k l:4 1 1). The main characteristics of YZn12 are: a (Å): 8.8750, b (Å): 8.8750, c (Å): 5.1920, Alpha (°): 90.0, Beta (°): 90.0, Gamma (°): 90.0, calculated density (g/cm^3^): 7.09, Volume of the cell (10^6^ pm^3^): 408.95. The spatial group of YZn12 is tetragonal (I4/mmm) with a three-dimensional structure [32,33]. A single Y atom is connected to 20 Zn atoms in a 12-coordinate spatial geometry. The Y–Zn links are positioned at different distances between 3.16–3.41Å. Here, there are three different positions of Zn, first Zn is connected in a denatured geometry to 2 similar Y and 10 Zn atoms. Besides Y-Zn bonds, there are also Zn–Zn links with distances from 2.60–2.88 Å. For the second Zn position, Zn atom is connected to two Y atoms and ten Zn atoms to form a composite of margin, face, and angle corner distribution of Zn in Y2Zn10 (with 2 links of 2.60 Å and 4 of 2.74 Å). For the third position, Zn is linked in a ten-coordinate geometry to one Y and 9 Zn atoms (Zn–Zn link length is 2.59 Å).

A Mg12ZnY intermetallic compound was also identified and exhibits excellent mechanical properties in Mg-based alloys [34]. It is known from the literature that the Mg12ZnY phase has a significant role in enhancing the mechanical properties of alloys [35,36,37]. This compound presents a hexagonal crystal structure [32] with lattice parameters (nm): a = 0.321 and c = 3.694. These compounds with Y were initially formed pending solidification stage and did not manage to dissolve completely into the α–Zn matrix. To obtain a supra-saturated α-Zn solid-solution, a hardened solution heat treatment is necessary, and to promote this type of precipitation, aging heat treatment at low temperature can be used for obtaining a fine lamellar structure [38]. A higher percentage of solid solution in the experimental alloys will promote a generalized corrosion at the contact with an electrolyte, and a higher number of intermetallic compounds will favor the micro-hardness increase.

### 3.2. Immersion Tests

Samples were analyzed after the immersion test (37 ± 1 °C, 10xDPBS) by weighing, and the surface by chemical composition acquisitions. Researchers in the field of biodegradable materials agree that the corroding environment has an important influence on the corrosion rate and the behavior of an alloy [39]. Similar to Mg-based alloys big differences were observed in the behavior of Zn-alloys in different electrolyte solutions like simulated body fluids, Hanks’s solution, Ringer, Dulbecco, or Dulbecco plus bovine serum [40]. For all samples, an increase in weight is observed after 24, 48, and 72 h (Table 3), except the Zn_3_Mg_0.5_Y alloy at 24 h, which presented a higher added mass than all other materials. The samples show an increase in mass with 1 to 2.6 mg thanks to reactions between materials and DPBS solution. Ultrasound cleaning in alcohol was made to remove unstable compounds from the surface after the immersion test. Only a small part of the compound form on the surface was detached and passed in solution, a fact that shows good stability of the layer formed on top of the alloy during the first three days of immersion. Further research will be conducted to establish the period of layer stability and the degradation starting point by mass loss perspective. When the compound layer formed on the surface with high participation of Zn and Mg will be detached, a further corrosion process will decrease the mass.

It has been proved countless times that electrolyte environment influences the degradation rate and the corrosion compound type. The corrosion rate is relatively lower in 10xDPBS than other mediums like simulated body fluids (SBF) [39]. This behavior is related to the differences in chemical composition, pH values, and different quantities of anorganic salts or aminoacids [41,42,43,44].

An important difference that Zn-based materials show, compared to Mg-based alloys, are the normal cathodic reactions that occur [39]:

2H_2_O + 2e^−^ → H_2_ + 2OH^−^ (1)

2H_2_O + O_2_ + 4e^−^ → 4OH^−^ (2)

The release of H_2_ quantity (reaction (1)) beside O_2_ reduction (reaction (2)) is much smaller for Zn alloys compared to Mg.

The corrosion Compounds have a partially protective role and decreased the corrosion rate. Mainly, the degradation of Zn is produced by the next anodic reaction (Equation (3)):

Zn→Zn^2+^ + 2e^−^ (3)

The compounds resulting from these reactions are normally ZnO or/and Zn(OH)_2_. The growth of Zn(OH)_2_ is more harmful based on his lower stability compared to ZnO. Moreover, Zn(OH)_2_ is dissolved in contact with chlorides Equation (4), enhancing the degradation process. In this study, the polarization resistance is increased in 10xDBPS solution with only 8 g/L NaCl.

Zn(OH)_2_ + 2 Cl^−^ →Zn^2+^ + 2 Cl^−^ + 2 OH^−^ (4)

In the case of Zn-based alloys, insoluble phosphates Equation (5), or hydrozincite (Zn_5_(CO_3_)_2_(OH)_6_) and simonkolleite (Zn_5_(OH)_8_Cl_2_⋅H_2_O) compounds are more numerous, which affects the general corrosion rate enhancing in the same time the polarization resistance of the material.

3 Zn^2+^ + 2 HPO_4_^2−^ + 2 OH^−^ + 2 H_2_O→Zn_3_(PO_4_)_2_ + 4 H_2_O (5)

**Figure 5:**
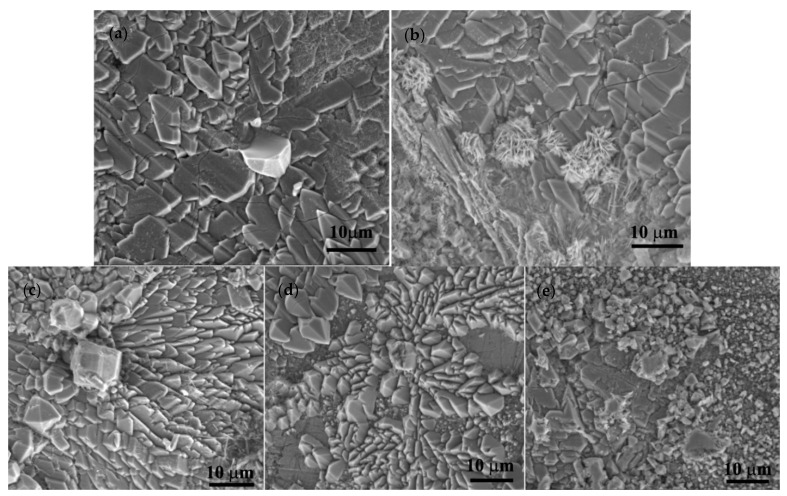
SEM after immersion period of three days and ultrasound cleaning: (**a**) Zn, (**b**) Zn_3_Mg, (**c**) ZnMg_0.4_Y, (**d**) ZnMg_0.5_Y, and (**e**) ZnMg_0.6_Y.

Generally, at the first contact, the corrosion rate is slower in 10xDPBS or similar solutions for all biodegradable metals (Mg– or Fe-base) [39]. The corrosion layer formed during immersion is uniform in DPBS and consists of oxides (mainly of Zn) and unsolvable corrosion products made through precipitation, such as metallic oxides/hydroxides, phosphates, and carbonates. The additional mass gained after the immersion tests observed in Table 3 is based on compound accumulation and higher corrosion resistance in the first days of contact with the electrolyte. Comparing the behavior of similar alloys in SBF, the slower corrosion rate can be attributed to the growth of a more stable compound layer, adsorbed amino-acids, and lack of HCl that accelerates corrosion in SBF [39]. The surface of the samples after immersion presents a manly covered area by compounds (Figure 5); most of them passed from the electrolyte solution, interacted with the surface, and produced a resistant corrosion layer. After three days, all samples showed a cover layer on the surface formed from Zn, O, and P (Table 4), small percentages of Mg, Y–oxides and Cl, K as salts from the solution. The morphological structure of the compounds is similar to Zn_3_(PO_4_)_2_ (O_8_P_2_Zn_3_) compound layer for all samples, a layer that covers almost the entire surface [45,46].

Salt compound morphology can be observed as ZnMgY deposits on the surface (Figure 5c, confirmed by Table 4) with Zn_3_(PO_4_)_2_ layer on top. The order of interactions is confirmed by the first formation of the phosphated layer and second by other compounds that passed from the electrolyte to surface. On the surface of Zn_3_Mg alloy appearance of MgO can be observed on the surface [47]. Uncovered areas were observed on all samples (Figure 5d), and many fine cracks define the deposited layer. The layer formed on the surface has a certain stability after three days of immersion, ultrasound cleaning only removed a part of the compounds (Table 3), but with further contact with the solution, the corrosion will break the phosphated layer, and the material will pass in electrolyte continuing the degradation. The stability of the material in the first three days of immersion can be considered an advantage for biomedical applications.

All surfaces presented a high percentage of O and P after three days of immersion. Small quantities of Cl and K are also identified. Depending on the stability and thickness of the phosphated layer formed on the surface, the Y element was also identified.

### 3.3. Potentiodynamic Polarization Curves

**Figure 6:**
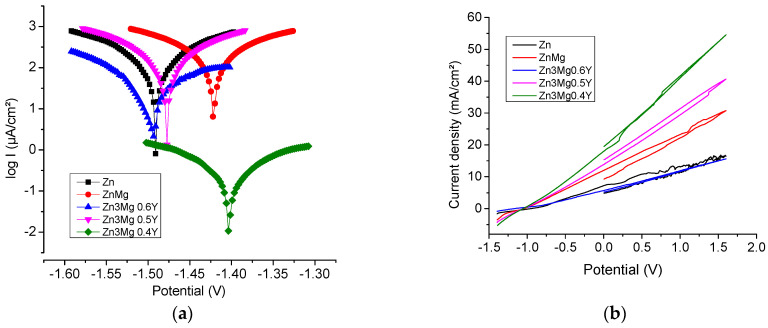
Linear and cyclic potentiometry of the samples. (**a**) Tafel diagram and (**b**) cyclic potentiometry.

The Linear polarization method was used to determine the corrosion rate of Zn, ZnMg with different percentages of Y in 10xDPBS electrolyte. Linear polarization curves are shown in Figure 6a and cyclic polarization curves in Figure 6b. Using electro-chemical polarization methods, we can provide information about the type of corrosion and anodic or cathodic protection. Linear and cyclic potentiometry techniques can be useful for evaluation analysis of the passive region, the passivation possibility of a material in contact with the environment, stability, and the passivation quality (degradation rate).

The corrosion parameters obtained from the Tafel extrapolation are listed in Table 5. Linear polarization curves show that the corrosion potential values (E_corr_) were similar with an increase in percentages of Y. This effect can be related to the presence of a passivation process of the surface, as in all metallic materials, and the passivation can last more or less, influencing the degradation rate and the material stability.

The tendency of corrosion current density (J_corr_) values is to decrease along with the increase in percentages of Y from 575.7 µA/cm^2^ to 66.55 µA/cm^2^. In the case of Zn3Mg_0.6_Y is observed a decrease in the current density (J_corr_) due to the formation of intermetallic Mg_2_Zn_11_ and MgZn_2_ fazes. The corrosion rate of Zn_3_Mg_0.4_Y is 6.46 mm/Y, much higher than Zn and Zn_3_Mg 3.36 mm/Y, and 2.85 mm/Y. This is due because the non-homogeneous structure caused by formation of new compounds with Y. A higher concentration of Y homogenizes the composition of the compounds and decreases the corrosion rate to 0.74 mm/Y.

The corrosion potential (E_corr_) for Zn_3_Mg_0.4_Y shifted by about 60 mV compared to Zn_3_Mg, indicating a diminution of the corrosion tendency in thermodynamics. A higher concentration of Y had no further significant impact on E_corr_.

From cyclic voltammograms presented in Figure 6b, the similar behavior of the alloys and uniform corrosion is observed, reflected by the sharp increase in current density. In the Zn_3_Mg_0.6_Y case, the cathodic branch of the voltammogram overlaps the anodic branch, indicating advanced corrosion resistance.

### 3.4. Micro-Hardness Characterization of the Samples

**Figure 7:**
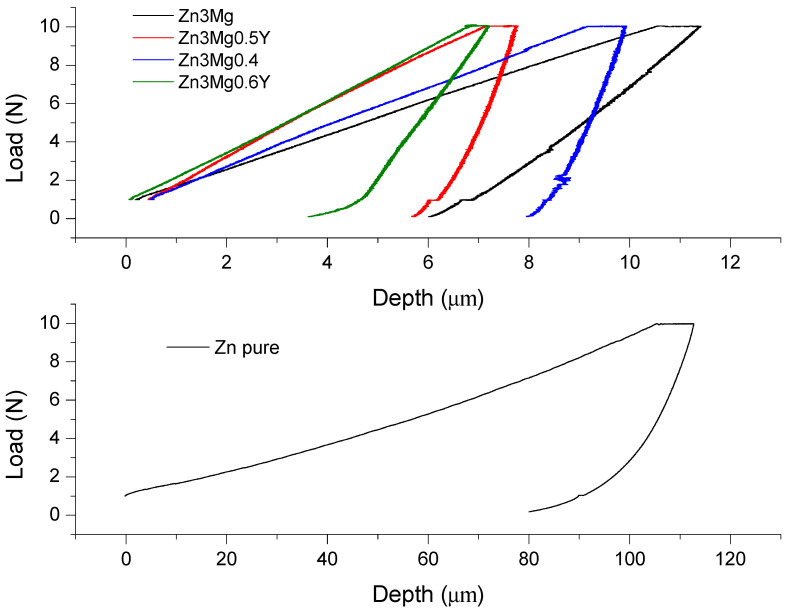
Microhardness experiments pure Zn and Zn-alloy behavior.

Experiments to establish the microhardness of the samples were realized in laboratory conditions [48,49]. Load-depth variation graphs were obtained in Origin 2021 software (OriginLab Corporation, Northampton, MA, USA). Rockwell microhardness determinations were realized on five areas of the samples. The big difference in the indentation behavior of Zn toward ZnMg and ZnMgY alloys determined us to provide two graphs in Figure 7, with different depth scales.

The microhardness of a material can give information about the material elongation to fracture, plasticity, and resistance to fracture of materials. The addition of magnesium to zinc clearly improves the microhardness of the material (7.21 times), especially through compounds and phases formed in the microstructure. Furthermore, the addition of the Y element increases even more the microhardness of the alloy till values of 1.25 GPa. The comparative values are presented in Table 6, with a standard deviation of the experimental values. The increase in microhardness with addition of Y element is due to the formation of new compounds YZn_12_ and Mg_12_ZnY. Microhardness seems to increase along with the Y content, but further research in this area is required for confirmation.

Indentation experiments can be useful to analyze the elastic modulus for various depths. From this test, more information about the material can be gathered in the same time based on the load and unload curve. We use average values from separate indentations made into a polycrystalline material and each indent can be situated in different positions regarding the solid solution, intermetallic, or other compounds.

The softer material is pure zinc (higher maximum displacement), and the opposite part is the value for Zn_3_Mg_0.5_Y, 6.88 µm. These compounds, based on Mg and Y, have areas under 5000 µm^2^ and a homogeneous spread. In almost every indentation case (see Table 6 for contact area values), the indenter tip presses also on a compound at least partially. In case of Zn3Mg_0.5_Y alloy, the higher values of indentation Young Modulus and microhardness are based on the fifth measurement point which, probably, was mostly on a harder compound. This affirmation was confirmed by scanning electron microscopy images. Young’s modulus values presented an increase with the addition of Mg and a bigger increase with Y, and the Zn_3_Mg_0.5_Y alloy presents the highest value of 81.37 GPa (32.42 times bigger than pure zinc Young modulus). The contact stiffness presents an increase with Y addition based on the increase in material hardness.

## 4. Conclusions

The effect of Y concentration on the ZnMg alloys properties was investigated. Analyzing the experimental results, we conclude that:

- The Mg and Y addition to pure Zn improves mechanical properties as microhardness, Young’s modulus, and contact stiffness.
- The appearance of YZn12 and Mg12ZnY intermetallic phases among ZnMg compounds has the most significant importance concerning the modification of Zn properties.
- The growth of the Zn3PO4 layer on the surface alloys provides protective corrosion resistance effect.
- Y participation in formation of YZn12 intermetallic phase elements gives the new alloy a more noble potential than pure Zinc.
- Formation of YZn12 intermetallic compound has been correlated with a more aggressive galvanic corrosion between Mg2Zn11 and YZn12 with the Zn matrix.

## Figures and Tables

**Table 1:** Chemical composition analysis of structural elements of ZnMgY alloy (Zn3Mg0.6Y).

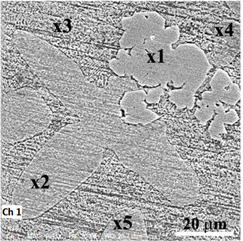	**Elements/Analysis Points**	**Zn**		**Mg**		**Y**	
		**wt%**	**at%**	**wt%**	**at%**	**wt%**	**at%**
	x1	89.11	91.76	–	–	10.88	8.24
	x2	99.2	98.4	0.52	1.4	0.28	0.2
	x3	96.28	92.16	2.79	7.19	0.93	0.65
	x4	99.12	99.19	0.08	0.22	0.8	0.59
	x5	99.43	98.48	0.57	1.52	–	–
	EDS err.	2.5		0.3		0.5	

**Table 2:** Chemical compositions of the samples (average values from five determinations).

Materials/Elements	Zn		Mg		Y	
	wt%	at%	wt%	at%	wt%	at%
Zn pure	99.99	99.99	−	−	−	−
Zn3Mg	97.02	92.63	2.98	7.37	−	−
Zn3Mg0.4Y	96.8	92.90	2.78	6.74	0.42	0.26
Zn3Mg0.5Y	96.75	92.96	2.75	6.59	0.50	0.35
Zn3Mg0.6Y	96.55	86.93	2.82	5.74	0.63	0.42
EDS error	1.1		0.4		0.1	

StDev: Zn: ±1.5, Mg: ±0.2 and Y: ±0.1.

**Table 3:** Weights of samples after the immersion test.

Samples		Initial Weight [g]	Weight After Immersion [g](mg)	Weight After Ultrasound Cleaning [g](mg)
Zn	24H	4.1888	4.1906 (+1.8)	4.1902 (+1.4/−0.4)
	48H	3.9416	3.9431 (+1.5)	3.9428 (+1.2/−0.3)
	72H	4.1369	4.1391 (+2.2)	4.1387 (+1.8/−0.4)
ZnMg	24H	2.6388	2.6410 (+2.2)	2.6401 (+1.3/−0.9)
	48H	3.4378	3.4394 (+1.6)	3.4386 (+0.8/−0.8)
	72H	2.4310	2.4328 (+1.8)	2.4326 (+1.6/−0.2)
Zn_3_Mg_0.4_Y	24H	2.2969	2.2981 (+1.2)	2.2979 (+1.0/−0.2)
	48H	2.9427	2.9437 (+1.0)	2.9434 (+0.7/−0.3)
	72H	1.9240	1.9266 (+2.6)	1.9258 (+1.8/−0.8)
Zn_3_Mg_0.5_Y	24H	2.4688	2.4755 (+6.7)	2.4753 (+6.5/−0.2)
	48H	2.5858	2.5870 (+1.2)	2.5869 (+1.1/−0.1)
	72H	2.2160	2.2174 (+1.4)	2.2167 (+0.7/−0.7)
Zn_3_Mg_0.6_Y	24H	2.2581	2.2605 (+2.4)	2.2604 (+2.3/−0.1)
	48H	2.2529	2.2548 (+1.9)	2.2547 (+1.8/−0.1)
	72H	2.1964	2.1981 (+1.7)	2.1976 (+1.2/−0.5)

**Table 4:** Chemical composition of the surface samples after immersion test (after 3 days and ultrasound cleaning).

Material	Zn		Mg		Y		O		P		Cl		K	
	wt%	at%	wt%	at%	wt%	at%	wt%	at%	wt%	at%	wt%	at%	wt%	at%
Zn pure	55.42	26.97	–	–	–	–	28.76	57.17	12.88	13.23	02.93	02.63	–	–
Zn3Mg	51.60	24.26	1.09	1.38	–	–	29.91	57.45	15.66	15.54	–	–	1.74	1.37
Zn3Mg0.4Y	51.29	23.96	0.67	0.85	0.25	0.09	30.65	58.51	15.70	15.48	–	–	1.44	1.12
Zn3Mg0.5Y	51.54	24.25	0.92	1.16	0.64	0.22	30.18	58.03	15.43	15.32	–	–	1.29	1.01
Zn3Mg0.6Y	53.25	26.80	0.55	0.74	01.36	00.50	25.05	51.50	17.26	18.33	–	–	2.53	2.13
EDS error	1.2		0.2		0.1		0.8		0.75		0.2		0.1	

StDev:Zn: ±1.74, Mg: ±0.41, Y: ±0.57, O: ±2.26, P:1.57, Cl: ±0, K: ±0.91.

**Table 5:** Linear potentiometry parameters.

Sample	−E_cor_ mV	b_a_ mV	b_c_ mV	R_p_ ohm.cm^2^	J_corr_ µA/cm^2^	V_corr_ mm/Y
Zn	1491.4	122.2	−141.1	168.97	210.09	3.36
Zn3Mg	1469.8	106.0	−132.0	167.01	253.8	2.85
Zn3Mg0.4Y	1402.3	134.5	−135.6	78.25	575.7	6.46
Zn3Mg0.5Y	1477.9	117.9	−129.1	146.06	311.6	3.49
Zn3Mg0.6Y	1497.0	133.9	−95.8	851.46	66.55	0.74

**Table 6:** Microhardness experiment values for samples.

Alloys		Indentation Young Modulus (GPa)	Hardness (GPa)	Contact Stiffness (N/µm)	Maximum Load (N)	Maximum Displacement (µm)	Contact Depth (µm)	Contact Area (µm^2^)
Zn pure	**Average**	2.51	0.14	0.79	8.96	65.48	57.29	7,8807.47
	**Std. Dev.**	0.51	0.05	0.05	0.02	26.59	26.86	5,3656.28
Zn_3_Mg	**Average**	17.10	1.01	1.95	9.01	10.80	7.31	9022.66
	**Std. Dev.**	1.73	0.06	0.22	0.01	0.46	0.49	599.36
Zn_3_Mg_0.4_Y	**Average**	59.64	0.79	7.23	8.79	9.91	8.95	1,1002.22
	**Std. Dev.**	12.95	0.04	1.47	0.37	0.49	0.43	507.04
Zn_3_Mg_0.5_Y	**Average**	81.37	1.22	7.94	9.01	6.88	6.03	7459.77
	**Std. Dev.**	10.01	0.16	0.69	0.02	0.70	0.69	841.89
Zn_3_Mg_0.6_Y	**Average**	39.26	1.25	3.77	9.02	7.51	5.70	7062.93
	**Std. Dev.**	1.34	0.11	0.36	0.01	0.31	0.35	430.67

## Data Availability

Data sharing not applicable.
